# Data on acoustic phonetic properties of non-coronal fricatives in monosyllabic words of Zhongjiang Chinese

**DOI:** 10.1016/j.dib.2022.108062

**Published:** 2022-03-16

**Authors:** Dongmei Rao, Jason Shaw, Rikker Dockum

**Affiliations:** a School of Literature, Journalism, and Communication, Xihua University, Chengdu, Sichuan Province, China; b Department of Linguistics, Yale University, New Haven, CT, USA; c Department of Linguistics, Swarthmore College, Swarthmore, PA, USA

**Keywords:** Acoustic phonetics, Fricatives, Chinese dialects, Sound change

## Abstract

The data reported in this article are non-coronal fricative measurements from 10 (5 male; 5 female) native speakers of Zhongjiang Chinese. Each speaker produced 10 repetitions of 90 monosyllabic words beginning with either a velar fricative, /x/, or a labial-dental fricative, /f/. The measurements reported include spectral properties often used to characterize fricative variation, including: spectrum center of gravity (CoG), spectrum standard deviation (SD), spectrum skew, spectrum kurtosis, maximum amplitude frequency, and maximum amplitude. These measurements are compared across two data filtering conditions: a high pass filter condition, in which a 300Hz high pass filter was applied to the data before spectral measurements were calculated, and a no filter condition. The 90 monosyllabic words include the target fricatives in different phonetic environments. Target words include some that historically derive from different fricatives and show variation across regional varieties of Mandarin Chinese. Subsets of the target materials enable several closely matched comparisons of items. We describe measurements across the whole dataset, comparing as well the effect that filtering has on the measurements. The data also include a CSV file with measurements of each token, which enables comparison of phonetic contexts, lexical effects and individual differences in fricative variation beyond those described here. For further discussion of the data, please refer to the full length article entitled “The role of gestural timing in non-coronal fricative mergers in Southwestern Mandarin: acoustic evidence from a dialect island. *Journal of Phonetics*” [6].

**Specifications Table**

**Table:** 

Subject	Linguistics
Specific subject area	Phonetics, dialect variation, and sound change
Type of data	Tables, figures, spreadsheet
How data were acquired	Acoustic measurements based on speech recorded in a studio setting.
Data format	Analysed
Parameters for data collection	Native speakers of the Zhongjiang dialect of Chinese were selected for participation. To minimize environmental noise, participants were recorded in a sound-attenuated room. Data were recorded at a high sampling rate to allow analysis of higher frequencies (greater than 8 KHz) characteristic of fricatives.
Description of data collection	The data in this paper were collected as part of a longer recording session, following standard protocol for naturalistic speech data elicitation.
Data source location	Institution: onsite fieldwork City/Town/Region: Zhongjiang City, Sichuan Province Country: China Latitude and longitude (and GPS coordinates, if possible) for collected samples/data: Longitude 104.67861, latitude 31.03297
Data accessibility	The raw data are hosted in a public repository. Repository name: OSF Data identification number: DOI 10.17605/OSF.IO/MJH46 Direct URL to data: https://osf.io/mjh46/
Related research article	D. Rao, J.A. Shaw, The role of gestural timing in non-coronal fricative mergers in Southwestern Mandarin: acoustic evidence from a dialect island, Journal of Phonetics 89 (2021), 101112.

**Value of the Data**

• Data reveal patterns of synchronic variation in fricative production that can be related to variation and change in language communities across Southwest China.
• Data are useful to phoneticians studying speech production and to historical linguists, sociolinguists and linguistic anthropologists studying language variation and change in China.
• Data can be used to test new hypotheses about non-coronal fricative variation, including which phonetic dimensions differentiate velar and labiodental fricatives and how these dimensions vary across contexts.
• Data provide a historical record of synchronic speech variation in Zhongjiang China, a dialect island, which may be in the process of assimilating under the influence of other language varieties.
• Data can be used to evaluate the effect of a high pass filter on common fricative measurements.

## 1 Data Description

The data presented in this article illustrate phonetic properties of the velar and labiodental fricatives in Zhongjiang Chinese. Some studies reporting spectral properties of fricatives apply a high pass filter to the acoustics before extracting measurements [1] while others report no such filter [2]. Some common phonetic measurements of fricatives, particularly, spectral moments [3] are particularly sensitive to whether the data has been high pass filtered, which can make these measurements difficult to compare across studies employing different filtering techniques, including no high pass filter. Other types of spectral measurements, such as the maximum frequency in the signal or the amplitude of the maximum frequency, which are also used to describe fricatives [4,5], are likely robust to filtering. This article presents spectral measurements with and without a high pass filter at 300 Hz, illustrating the sensitivity to this filter of phonetic measurements commonly used to describe fricatives.

The figures below compare measurements with and without the filter, across the entire dataset. The csv file submitted with the article, described in the supplementary materials section, is coded for specific subsets of the data designed to address different aspects of the pattern (see [6]). In addition to the csv file containing measurements of all tokens, the supplementary materials include a Praat [7] script for extracting the measurements with and without the filter and an R [8] script for outlier extraction and figure generation.

### 1.1 Data overview

**Fig. 1:**
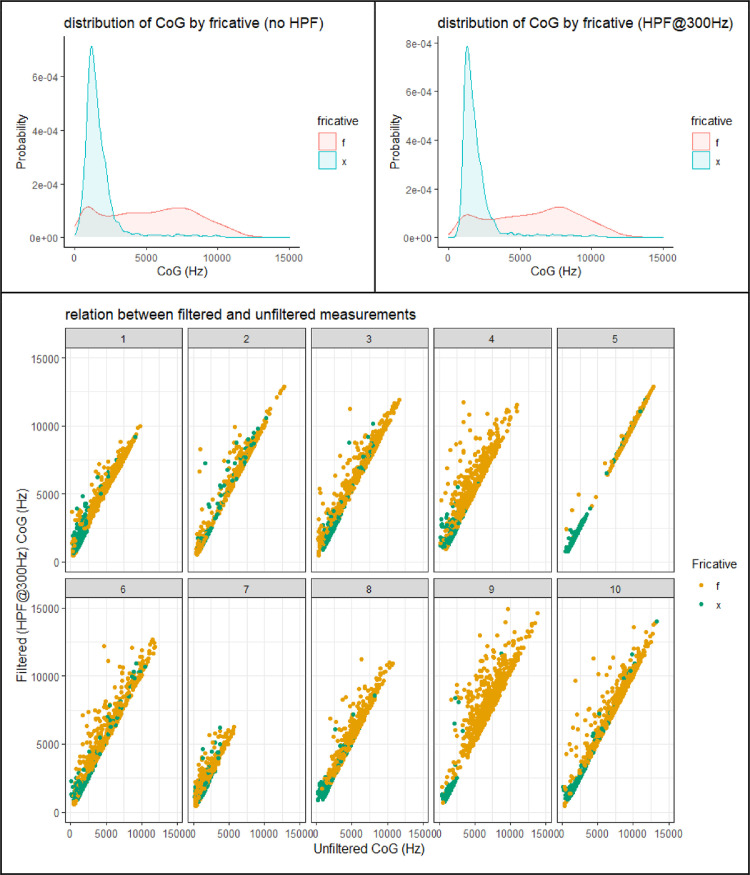
Comparison of filtered and unfiltered CoG measurements. Top panels show kernel density plots of spectrum CoG by fricative; the bottom panel plots unfiltered CoG (x-axis) against filtered CoG (y-axis).

**Fig. 2:**
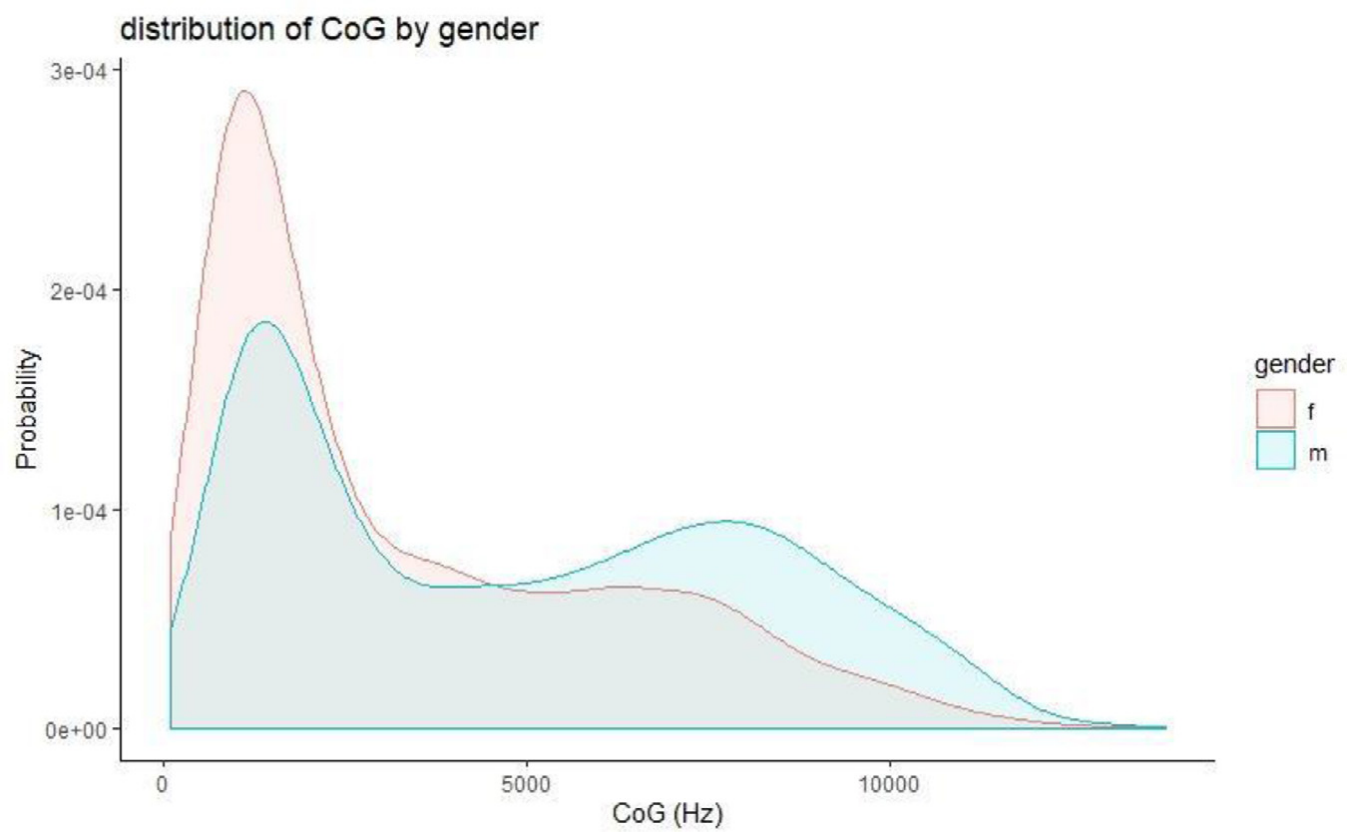
Kernel density plots of spectrum Center of Gravity (unfiltered) by gender.

**Fig. 3:**
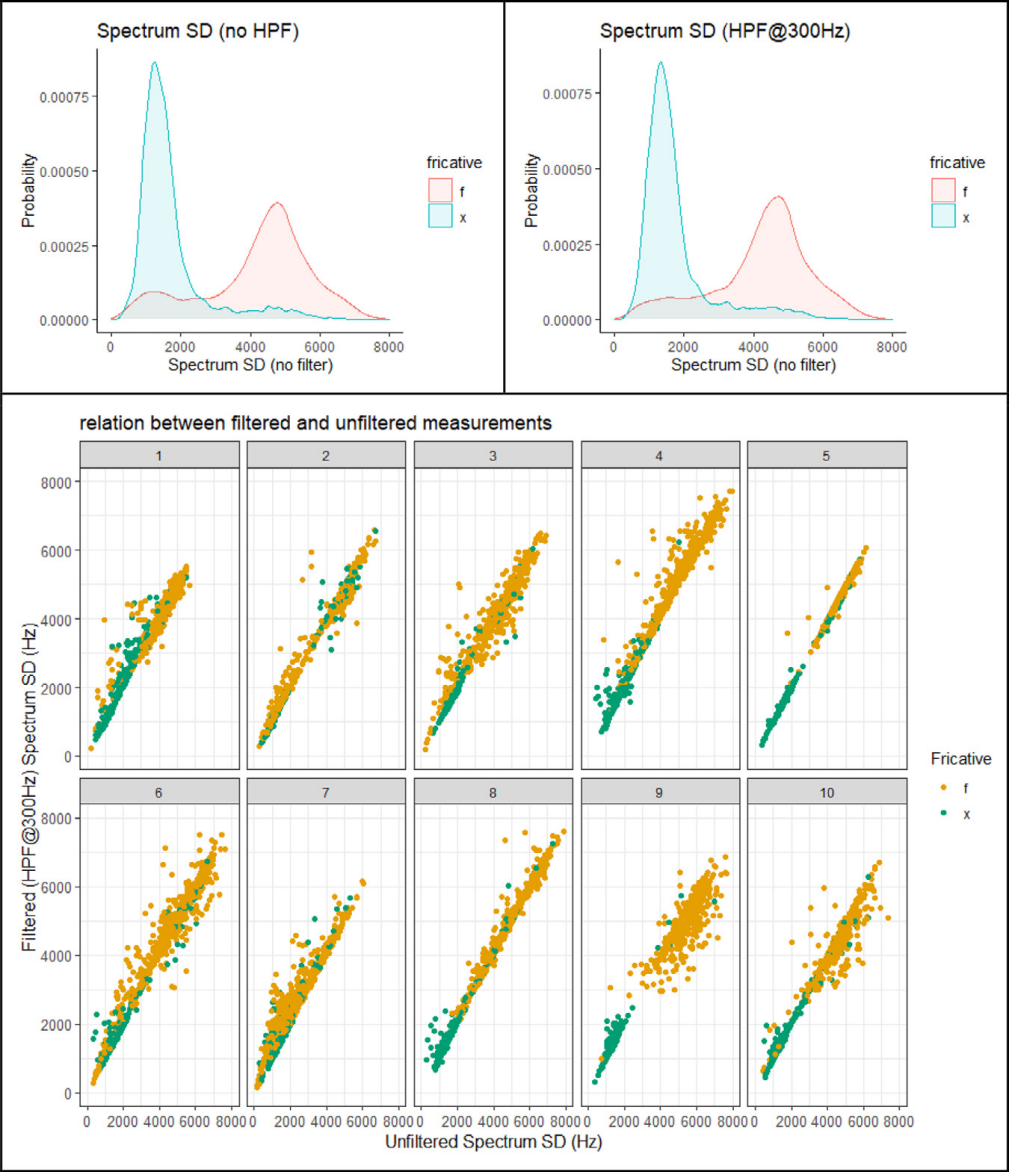
Comparison of filtered and unfiltered spectrum Standard Deviation (SD) measurements. Top panels show kernel density plots of spectrum CoG by fricative; the bottom panel plots unfiltered SD(x-axis) against filtered SD(y-axis).

**Fig. 4:**
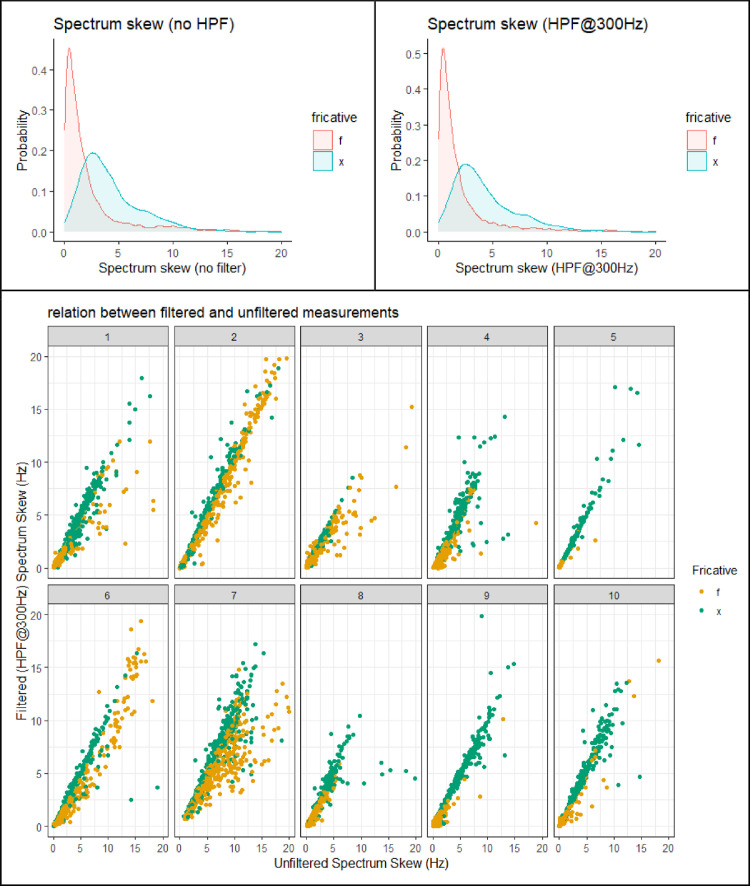
Comparison of filtered and unfiltered spectrum skew measurements. Top panels show kernel density plots of spectrum skew by fricative; the bottom panel plots unfiltered skew(x-axis) against filtered skew (y-axis).

**Fig. 5:**
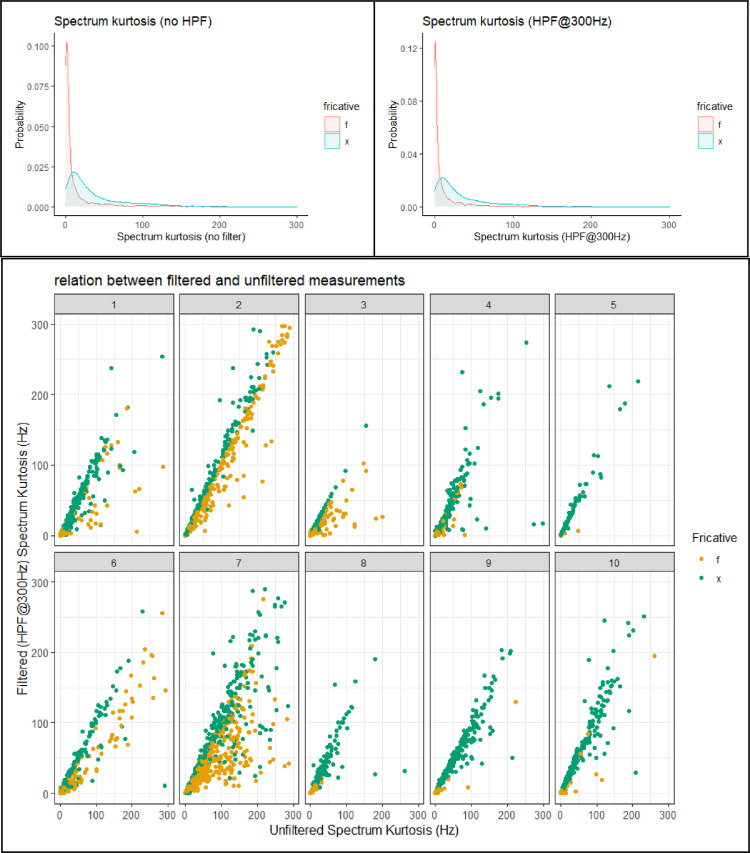
Comparison of filtered and unfiltered spectrum kurtosis measurements. Top panels show kernel density plots of spectrum kurtosis by fricative; the bottom panel plots unfiltered kurtosis (x-axis) against filtered kurtosis (y-axis).

**Fig. 6:**
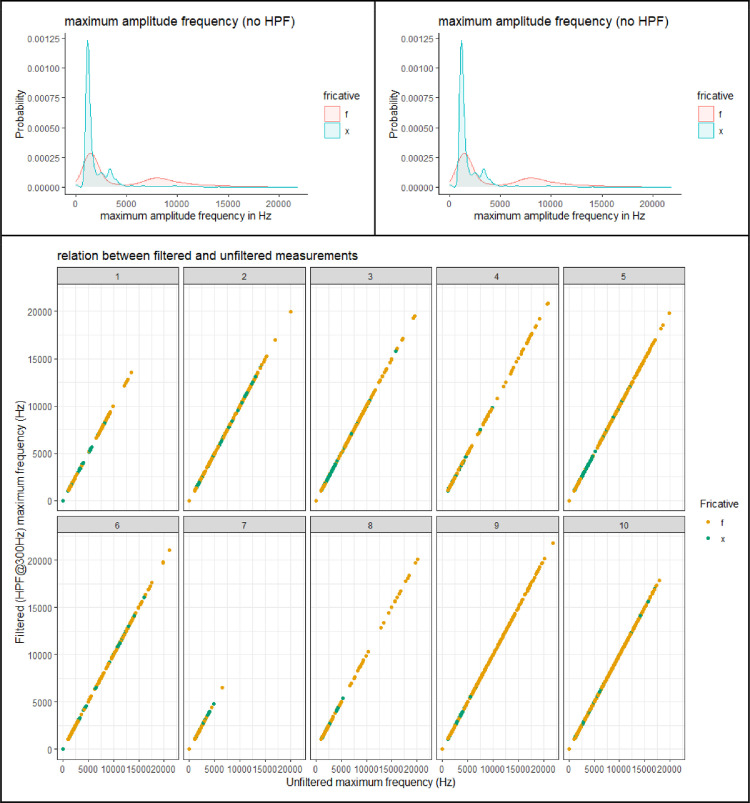
Comparison of filtered and unfiltered measurements of the maximum frequency in the spectrum. Top panels show kernel density plots by fricative; the bottom panel plots unfiltered maximum frequency (x-axis) against filtered maximum frequency (y-axis).

**Fig. 7:**
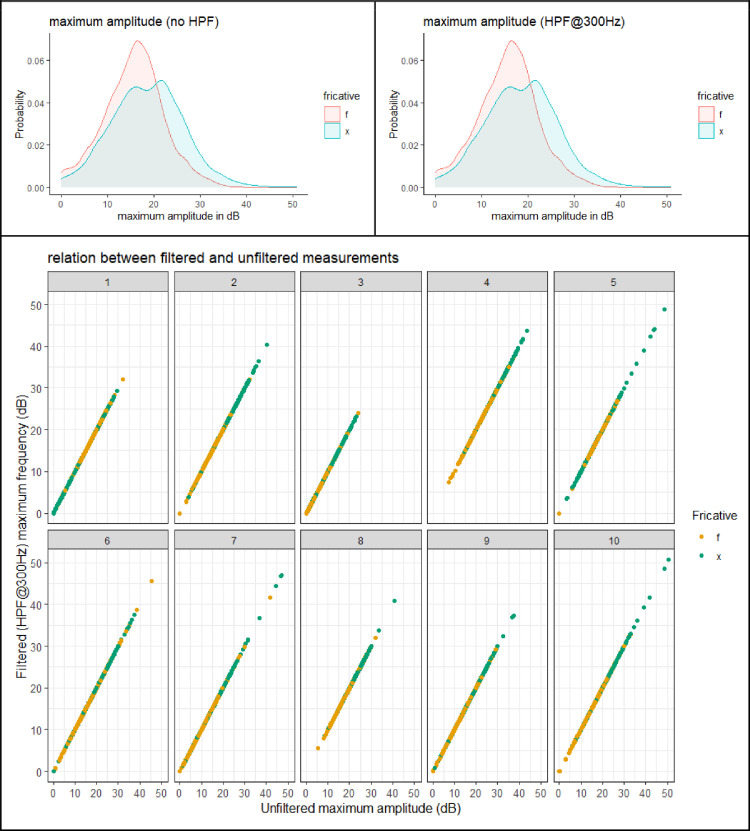
Comparison of filtered and unfiltered measurements of the maximum amplitude in the spectrum. Top panels show kernel density plots by fricative; the bottom panel plots unfiltered amplitude (x-axis) against filtered amplitude (y-axis).

This section presents figures of six spectral measurements of non-coronal fricatives, spectrum center of gravity (CoG), Fig. 1, spectrum standard deviation (SD), Fig. 3, spectrum skew, Fig. 4, spectrum kurtosis, Fig. 5, maximum amplitude frequency, Fig. 6, maximum amplitude, Fig. 7. For comparison, each measure is presented with and without a 300Hz high pass filter. The figures show that the filter impacts the measurements relative to spectral moments, including CoG, SD, skew and kurtosis, but not maximum amplitude frequency or maximum amplitude. We also present CoG by speaker gender in Fig. 2.

Fig. 1 compares spectrum Center of Gravity (CoG), with and without a high pass filter at 300 Hz. The top panels show the distribution of CoG values by fricative. The distribution for the /x/ fricative is centered at low frequency. Most of the values are below 5,000 Hz, regardless of filtering. The /f/ fricative shows a wider range of variation. The distribution for /f/ is bimodal, with a peak at high frequency, approximately 7,000 Hz, and another at low frequency, overlapping with the /x/ distribution. The high pass filter has the effect of raising CoG measurements somewhat. This can be seen in the kernal density plots in the top panels as well as the scatter plot in the bottom panel. The bottom panel plots the unfiltered CoG measurements (x-axis) against the filtered measurements (y-axis), by speaker. Since there is no probability mass below 300 Hz in the filtered data, the CoG values are shifted upwards, although the degree of the shift varies across both speakers and fricatives. Tokens that fall on the diagonal are unaffected by the filter. Tokens above the diagonal have a higher CoG in the filtered data than in the unfiltered data. The degree of this difference is variable, but appears to be larger for /f/ than for /x/.

Fig. 2 shows the distribution of CoG by gender across the entire data set, both /f/ and /x/ tokens. The distribution of CoG values for both genders is bimodal. The lower frequency mode is centered on a similar frequency across genders. The higher frequency mode shows some gender differences—the peak is higher frequency for males than for females.

Fig. 3 compares spectrum Standard Deviation (SD) across the entire dataset with and without the high pass filter. The top panels show density plots by fricative. The /x/ fricative has a low SD. The distribution of SD for /f/ is bimodal, at least in the unfiltered data (top, left panel). The larger peak of the distribution is centered around 4,500 Hz. The smaller peak of the /f/ distribution overlaps with /x/. The effect of filtering on spectrum SD varies across token, as can be seen in the lower panel, which plots unfiltered SD (x-axis) against filtered SD (y-axis) by speaker. For some speakers, filtering has the effect of raising spectrum SD. This is most clear for S07 and, to a lessor degree, S01 and S03. Filtering energy at low frequencies can increase the imbalance in amplitude across the spectrum. However, some tokens show decreased SD with filtering. This is the tendency, in particular, for S09 and S10. Thus, unlike CoG, the effect that filtering has on SD is not uniform across speakers. It depends in part on the speaker-specific distribution of energy across the spectrum.

Fig. 4 shows the spectrum skew by fricative. The distribution for both fricatives is mono-modal. The /f/ shows a lower and sharper peak in its distribution than /x/. The effect of the filter on this measurement is somewhat inconsistent. There is an overall tendency for the filter to lower skew, particularly for /f/. This is indicated by the number of tokens below the diagonal in scatter plot shown in the bottom panel of Fig. 4. However, there are also some tokens that are above the diagonal, indicating a raising effect of the high pass filter on skew.

Fig. 5 shows spectrum kurtosis by fricative. Kurtosis is similar for /f/ and /x/, but the distribution of values is more consistently low for /f/ than for /x/, as indicated by the density plots. The wider range of kurtosis values for /x/ is particularly salient for speakers 8, 9, and 10 in the by speaker scatter plot (Fig. 5: bottom). The effect of filtering on kurtosis measures is typically to lower kurtosis; however, in some tokens filtering increases kurtosis.

Fig. 6 shows the maximum amplitude frequency in the spectrum by fricative, with and without a 300 Hz high pass filter. The top panels show density plots and the bottom panel shows a scatter plot. The data points in the scatter plot fall on a straight line of slope 1, indicating that there is no effect of filter this measurement whatsoever. In terms of the difference in frequency by fricative, /x/ has a lower maximum frequency, typically around 2,000 Hz, with some higher values, likely due to individual differences. The maximum amplitude frequency for /f/, on the other hand, shows a bimodal distribution, with one mode around 8,000 Hz and the other overlapping heavily with /x/. This variation is also found in CoG measurements (Figure 1), which are discussed extensively in [6].

Fig. 7 shows the amplitude of the highest amplitude frequency by fricative with and without a high pass filter. As with the maximum amplitude frequency, there was no effect of filtering the data on the maximum amplitude measurement. This is illustrated by the identical distribution in the top panels and by the scatter plot on the bottom panel. There was a small effect of fricative. On average, the maximum amplitude for /x/ is somewhat greater than for /f/.

Fig. 1, Fig. 2, Fig. 3, Fig. 4, Fig. 5, Fig. 6, Fig. 7, presented as an overview of the data, display a subset of the measurements. The figures present key spectral measurements at the fricative midpoint. The complete set of measurements, including those made at earlier and later timepoints in the fricatives, are available in the supplementary data file. The original sound files and textgrids are available on OSF [9].

### 1.2 Supplementary data

The supplementary data file includes the measurements reported above (spectrum CoG, spectrum SD, spectrum skew, spectrum kurtosis, maximum amplitude frequency, maximum amplitude) in Fig. 1, Fig. 2, Fig. 3, Fig. 4, Fig. 5, Fig. 6, Fig. 7, sampled at different temporal intervals within the fricative. Measurements of fricative duration are also included in the data. The data are also coded for specific data subsets, described in [6], as well as for vowel context, the category of the fricative (/x/, /f/), the tone of the syllable, and the proto-category of each fricative, according to reconstructions of Middle Chinese. This coding facilitates construction of additional subsets to further explore variation in the data. The data is coded for speaker and for speaker gender. Additional information about the speakers, including age and occupation is not coded in the data file but is provided in the description of the speakers in the methods below (see Table 1). Columns containing measurements calculated without the high pass filter end in “nf” for “no filter”.

A description of the columns in the data file is as follows

(1) filename—the filename of the wav file for the token; in the OSF arhcive, each token is a separate file.
(2) syllable—unique number 1-90 for each item in the study; all items were monosyllabic. The syllable ID number corresponds to the first column in Table 2 (below).
(3) speaker—unique number 1-10 for each speaker in the study. The speaker number corresponds to the first column of Table 1 below.
(4) gender—binary gender of the speaker, coded as “f” or “m”.
(5) rep—the repetition number of the token, 1-10.
(6) pinyin—Standard Mandarin romanized orthography for the item.
(7) syllable_IPA—expected pronunciation of the item in the Zhongjiang variety; consonants, vowels, and tones are represented using the International Phonetic Alphabet. Tones are represented as Chao numerals where: 45 is a high-rising tone; 31 is a mid-falling tone; 51 is a falling tone; 324 is a falling-rising tone.
(8) fricative_IPA—coded as either “x” or “f” for the initial fricative that is expected to be produced in the word based on the description of Zhongjiang as a Type II merger variety of Southwest Mandarin; for more description of Mandarin merger types, see [6].
(9) rime_IPA—the syllable rime, including vowel, tone, and coda consonant, where present, in the International Phonetic Alphabet.
(10) Vowel_IPA—vowel in the International Phonetic Alphabet.
(11) tone_IPA—provides the Chao numeral for each lexical tone.
(12) tone_number—a single digit number, 1-4, representing the lexical tones, where: 1 is the high-rising tone; 2 is the mid-falling tone; 3 is the falling tone; and 4 is the falling-rising tone.
(13) study1_items—indicates whether the item was included in the study1 subset, coded as “0” (not included) or “1” (included).
(14) study2_items—indicates whether the item was included in the study2 subset, coded as “0” (not included) or “1” (included).
(15) study3_items—indicates whether the item was included in the study3 subset, coded as “0” (not included) or “1” (included).
(16) study4_items—indicates whether the item was included in the study4 subset, coded as “0” (not included) or “1” (included).
(17) proto_category—indicates the category of the initial fricative in Middle Chinese.
(18) max_amp_nf—the maximum amplitude of the highest amplitude frequency of the fricative spectrum sampled at the middle 20 ms of the fricative. This value was calculated without first high pass filtering the data. “nf” stands for “no filter”.
(19) max_freq_nf—the highest amplitude frequency of the fricative spectrum sampled at the middle 20 ms of the fricative, without first high pass filtering the data.
(20) COG_mid_nf—the spectrum center of gravity calculated at the middle 20 ms of the fricative, without first high pass filtering the data.
(21) SD_mid_nf—the spectrum standard deviation calculated at the middle 20 ms of the fricative, without first high pass filtering the data.
(22) Skew_mid_nf— the spectrum skew calculated at the middle 20 ms of the fricative, without first high pass filtering the data.
(23) Kurtosis_mid_nf— the spectrum kurtosis calculated at the middle 20 ms of the fricative, without first high pass filtering the data.
(24) COG1_nf—the spectrum center of gravity calculated at the first 20 ms of the fricative, without first high pass filtering the data.

**Table 1:** Gender, age, and occupation by speaker ID.

Speaker	Gender	Age	Occupation
1	M	61	factory worker
2	F	55	hospital nurse
3	M	62	retired elementary school teacher
4	F	56	retired elementary school teacher
5	M	60	factory worker
6	F	60	factory worker
7	F	68	retired elementary school teacher
8	M	65	retired elementary school teacher
9	M	61	city hall office worker
10	F	59	businessman

**Table 2:** List of the words recorded.

Syllable ID Number	Simplified Chinese Characters	Pinyin (standard romanized orthography)	Standard Mandarin IPA	Expected Zhongjiang IPA	Data Subset
1	法	fǎ	[fa^214^]	[fa^31^]	
2	发 (发财)	fā	[fa^55^]	[fa^31^]	Study 2(1)
3	付	fù	[fu^51^]	[fu^324^]	
4	喝	hē	[xɤ^55^]	[xo^45^]	
5	画	huà	[x^w^a^51^]	[fa^324^]	
6	肥	féi	[fei^35^]	[fei^31^]	Study 2(2)
7	黑	hēi	[xei^55^]	[xe^31^]	Study 3(3)
8	后	hóu	[xəu^51^]	[xəɯ^324^]	
9	放	fàng	[faŋ^51^]	[faŋ^324^]	
10	黄	huáng	[x^w^aŋ^35^]	[faŋ^31^]	
11	红	hòng	[xoŋ^35^]	[xoŋ^31^]	Study 4(3)
12	烦	fán	[fan^35^]	[fæ̃^31^]	Study 2(6)
13	混	hùn	[x^w^ən^51^]	[fən^324^]	
14	分	fēn	[fən^55^]	[fən^45^]	
15	飞	fēi	[fei^55^]	[fei^45^]	Study 2(4)
16	怀	huái	[x^w^ai^35^]	[fai^31^]	Study 3(4)
17	范	fàn	[fan^51^]	[fæ̃^324^]	
18	或	huó	[x^w^o^51^]	[fe^31^]	Study 3(3)
19	还 (还有)	hái	[xai^35^]	[xai^31^]	Study 3(4)
20	华	huá	[x^w^a^35^]	[fa^31^]	Study 2 (1)
21	罚	fá	[fa^35^]	[fa^31^]	
22	还 (还钱)	huán	[x^w^an^35^]	[fæ̃^31^]	Study 2 (6)
23	否	fǒu	[fəu^214^]	[fəɯ^51^]	Study 1(2)
24	方	fāng	[faŋ^55^]	[faŋ^45^]	
25	何	hé	[xɤ^35^]	[xo^31^]	
26	慌	huāng	[x^w^aŋ^55^]	[faŋ^45^]	
27	洪	hòng	[xoŋ^35^]	[xoŋ^31^]	Study 4(2)
28	回	huí	[x^w^ei^35^]	[fei^31^]	Study 2(2)
29	服	fú	[fu^35^]	[fu^31^]	
30	核	hé	[xɤ^35^]	[xe^31^]	
31	厚	hóu	[xəu^51^]	[xəɯ^324^]	
32	害	hài	[xai^51^]	[xai^324^]	Study 3(2)
33	划	huà/huá	[x^w^a^51^]/[x^w^a^35^]	[fa^324^]/[fa^31^]	
34	伐	fá	[fa^35^]	[fa^31^]	
35	合	hé	[xɤ^35^]	[xo^31^]	
36	房	fáng	[faŋ^35^]	[faŋ^31^]	
37	粪	fèn	[fən^51^]	[fən^324^]	
38	灰	huī	[x^w^ei^55^]	[fei^45^]	Study 2(4)
39	冯	féng	[fəŋ^35^]	[foŋ^31^]	Study 4(2)
40	号	hào	[hau^51^]	[xaɯ^324^]	
41	返	fǎn	[fan^214^]	[fæ̃^51^]	Study 2(7)
42	吼	hǒu	[xəu^214^]	[xəɯ^51^]	Study 1(2)
43	复	fù	[fu^51^]	[fu^31^]	
44	盒	hé	[xɤ^35^]	[xo^31^]	
45	昏	hūn	[x^w^ən^55^]	[fən^45^]	
46	费	fèi	[fei^51^]	[fei^324^]	
47	乏	fá	[fa^35^]	[fa^31^]	
48	浮	fú	[fu^35^]	[fəɯ^31^]/[fu^31^]	Study 1(1)
50	宏	hòng	[xoŋ^35^]	[xoŋ^31^]	
51	话	huà	[x^w^a^51^]	[fa^324^]	
52	翻	fān	[fan^55^]	[fæ̃^45^]	Study 2(5)
53	恢	huī	[x^w^ei^55^]	[fei^45^]	
54	婚	hūn	[x^w^ən^55^]	[fən^45^]	
55	废	fèi	[fei^51^]	[fei^324^]	
56	府	fǔ	[fu^214^]	[fu^51^]	
57	候	hóu	[xəu^51^]	[xəɯ^324^]	
58	花	huā	[x^w^a^55^]	[fa^45^]	Study 3(1)
59	缝 (缝纫)	féng	[fəŋ^35^]	[foŋ^31^]	Study 4(3)
60	欢	huān	[x^w^an^55^]	[fæ̃^45^]	Study 2(5)
61	坟	fén	[fən^35^]	[fən^31^]	
62	鸿	hòng	[xoŋ^35^]	[xoŋ^31^]	
63	芳	fāng	[faŋ^55^]	[faŋ^45^]	
64	父	fù	[fu^51^]	[fu^324^]	
65	晃	huàng	[x^w^aŋ^51^]	[faŋ^51^]	
66	封	fēng	[fəŋ^55^]	[foŋ^45^]	
67	豪	háo	[xau^35^]	[xaɯ^31^]	
68	荷	hé	[xɤ^35^]	[xo^31^]	
69	肺	fèi	[fei^51^]	[fei^324^]	Study 2(3)
70	饭	fàn	[fan^51^]	[fæ̃^324^]	Study 2(8)
71	猴	hòu	[xəu^35^]	[xəɯ^31^]	Study 1(1)
72	蜂	fēng	[fəŋ^55^]	[foŋ^45^]	
73	缓	huǎn	[x^w^an^214^]	[fæ̃^51^]	Study 2(7)
74	耗	hào	[xau^51^]	[xaɯ^324^]	
75	防	fáng	[faŋ^35^]	[faŋ^31^]	
76	轰	hōng	[xoŋ^55^]	[xoŋ^45^]	Study 4(1)
77	奋	fèn	[fən^51^]	[fən^324^]	
78	魂	hún	[x^w^ən^35^]	[fən^31^]	
79	粉	fěn	[fən^214^]	[fən^51^]	
80	谎	huǎng	[x^w^aŋ^214^]	[faŋ^51^]	
81	荤	hūn	[x^w^ən^55^]	[fən^45^]	
82	汇	huì	[x^w^ei^51^]	[fei^324^]	
83	环	huán	[x^w^an^35^]	[fæ^^~^^31]	
84	风	fēng	[fəŋ^55^]	[foŋ^45^]	Study 4(1)
85	哈	hā	[xa^55^]	[xa^45^]	Study 3(1)
86	海	hǎi	[xai^214^]	[xai^51^]	
87	好	hǎo	[xau^214^]	[xaɯ^51^]	
88	坏	huài	[x^w^ai^51^]	[fai^324^]	Study 3(2)
89	会	huì	[x^w^ei^51^]	[fei^324^]	Study 2(3)
90	换	huàn	[x^w^an^51^]	[fæ^^~^324^]	Study 2(8)
(25) SD1_nf—the spectrum standard deviation calculated at the first 20 ms of the fricative, without first high pass filtering the data.
(26) Skew1_nf— the spectrum skew calculated at the first 20 ms of the fricative, without first high pass filtering the data.
(27) Kurtosis1_nf— the spectrum kurtosis calculated at the first 20 ms of the fricative, without first high pass filtering the data.
(28) COG2_nf—the spectrum center of gravity calculated at the second 20 ms of the fricative, without first high pass filtering the data.
(29) SD2_nf—the spectrum standard deviation calculated at the second 20 ms of the fricative, without first high pass filtering the data.
(30) Skew2_nf— the spectrum skew calculated at the second 20 ms of the fricative, without first high pass filtering the data.
(31) Kurtosis2_nf— the spectrum kurtosis calculated at the second 20 ms of the fricative, without first high pass filtering the data.
(32) COG3_nf—the spectrum center of gravity calculated at the penultimate 20 ms of the fricative, without first high pass filtering the data.
(33) SD3_nf—the spectrum standard deviation calculated at the penultimate 20 ms of the fricative, without first high pass filtering the data.
(34) Skew3_nf— the spectrum skew calculated at the penultimate 20 ms of the fricative, without first high pass filtering the data.
(35) Kurtosis3_nf— the spectrum kurtosis calculated at the penultimate 20 ms of the fricative, without first high pass filtering the data.
(36) COG4_nf—the spectrum center of gravity calculated at the final 20 ms of the fricative, without first high pass filtering the data.
(37) SD4_nf—the spectrum standard deviation calculated at the final 20 ms of the fricative, without first high pass filtering the data.
(38) Skew4_nf— the spectrum skew calculated at the final 20 ms of the fricative, without first high pass filtering the data.
(39) Kurtosis4_nf— the spectrum kurtosis calculated at the final 20 ms of the fricative, without first high pass filtering the data.
(40) StartTime—the time stamp of the start of the fricative, in seconds.
(41) EndTime— the time stamp of the end of the fricative, in seconds.
(42) C_dur—the duration of the fricative, in milliseconds (ms).
(43) mid_start—the time stamp of the start of the middle 20 ms window, in seconds.
(44) mid—the time stamp of the middle of the fricative, in seconds.
(45) mid_end—the time stampe of the end of the middle 20 ms window, in seconds.
(46) COG_mid—the spectrum center of gravity calculated at the middle 20 ms of the fricative, after high pass filtering the data.
(47) SD_mid—the spectrum standard deviation calculated at the middle 20 ms of the fricative, after high pass filtering the data.
(48) Skew_mid— the spectrum skew calculated at the middle 20 ms of the fricative, after high pass filtering the data.
(49) Kurtosis_mid— the spectrum kurtosis calculated at the middle 20 ms of the fricative, after high pass filtering the data.
(50) max_amp—the maximum amplitude of the highest amplitude frequency of the fricative spectrum sampled at the middle 20 ms of the fricative, after high pass filtering the data.
(51) max_freq—the highest amplitude frequency of the fricative spectrum sampled at the middle 20 ms of the fricative, after high pass filtering the data.
(52) COG1_nf—the spectrum center of gravity calculated at the first 20 ms of the fricative, after high pass filtering the data.
(53) SD1_nf—the spectrum standard deviation calculated at the first 20 ms of the fricative, after high pass filtering the data.
(54) Skew1_nf— the spectrum skew calculated at the first 20 ms of the fricative, after high pass filtering the data.
(55) Kurtosis1_nf— the spectrum kurtosis calculated at the first 20 ms of the fricative, after high pass filtering the data.
(56) COG2_nf—the spectrum center of gravity calculated at the second 20 ms of the fricative, after high pass filtering the data.
(57) SD2_nf—the spectrum standard deviation calculated at the second 20 ms of the fricative, after high pass filtering the data.
(58) Skew2_nf— the spectrum skew calculated at the second 20 ms of the fricative, after high pass filtering the data.
(59) Kurtosis2_nf— the spectrum kurtosis calculated at the second 20 ms of the fricative, after high pass filtering the data.
(60) COG3_nf—the spectrum center of gravity calculated at the penultimate 20 ms of the fricative, after high pass filtering the data.
(61) SD3_nf—the spectrum standard deviation calculated at the penultimate 20 ms of the fricative, after high pass filtering the data.
(62) Skew3_nf— the spectrum skew calculated at the penultimate 20 ms of the fricative, after high pass filtering the data.
(63) Kurtosis3_nf— the spectrum kurtosis calculated at the penultimate 20 ms of the fricative, after high pass filtering the data.
(64) COG4_nf—the spectrum center of gravity calculated at the final 20 ms of the fricative, after high pass filtering the data.
(65) SD4_nf—the spectrum standard deviation calculated at the final 20 ms of the fricative, after high pass filtering the data.
(66) Skew4_nf— the spectrum skew calculated at the final 20 ms of the fricative, after high pass filtering the data.
(67) Kurtosis4_nf— the spectrum kurtosis calculated at the final 20 ms of the fricative, after high pass filtering the data.

## 2 Experimental Design, Materials and Methods

This section describes the experimental design and methods involved in developing a corpus of phonetic measurements of approximately 9,000 tokens of Zhongjiang Chinese non-coronal fricatives. Associated files include a spreadsheet of the measurements, the Praat scripts used to make the measurements, and an R script used for plotting the data, including some data preparation, coding and cleaning. The raw data, including wav files and segmentation (in the form of Praat Textgrids), are publicly available in a data repository [9].

### 2.1 Speakers

All of the speakers were born and raised in urban Zhongjiang. Gender, age, and occupation are given in Table 1.

### 2.2 Materials

The complete list of elicited words is provided in Table 2. There are a total of 90 monosyllabic words beginning with /x/ or /f/. The words in the table include these fricatives in different environments, including those predicted to condition fricative merger [6]. The data subsets that entered into each of the four studies in [6] are indicated in column 6. These data subsets consist of word pairs. The words that form pairs for each study are indicated in the final column, with an index grouping minimal pairs in parentheses. For example, syllable 2 is 发 (Standard Mandarin IPA [fa55], Zhongjiang IPA [fa31]) and was included in study 2, as indicated in the last column; it is paired in study 2 with syllable 20 华 (Standard Mandarin IPA [xwa35] Zhongjiang IPA [fa31]). Syllable 2 and syllable 20 are labelled as “Study2(1)” in the last column; the “(1)” indicates that they are the first minimal pair included in that study. In the accompanying data file, there are four columns which indicate which tokens were included in which studies. The columns are labelled: “Study1_items”, “Study2_items”, “Study3_items”, and “Study4_items”. Each row in these columns is coded as either “0”, if the token is not included in the study or “1”, if the token is included in the study.

### 2.3 Procedure

The word list above was recorded in a sound-attentuated environment as a part of a longer recording session. Details on the recording procedure are provided in [6]. Here we focus on a description of the data measurements.

The materials submitted with this paper include measurements from 8,991 wav files and corresponding Praat textgrids, indicating the segment boundaries for fricatives and vowels used to extract the measurements. The segment boundaries in the textgrids were determined by forced alignment, using the Montreal Forced Aligner [10], after first checking the aligner performance against 100 hand-segmented items. The original sound files and the textgrids based on a Zhongjiang-trained forced aligner as well as the textgrids based on hand segmentation are publicly available at [9].

A total of 9 tokens (0.1%) were excluded due to alignment failure. Of 9,000 tokens recorded, 8,991 are represented in the data file.

The measurements reported in the data file were extracted using Praat [7] with reference to the segment boundaries from forced alignment. Some spectral measurements were taken at five different timestamps in the target fricatives: the first 20 ms of the fricative, the second 20 ms of the fricative, the middle 20 ms of the fricative, the penultimate 20 ms time window and the final 20 ms of the fricative. The spectral measurements extracted at these time windows were: spectrum center of gravity (CoG), spectrum standard deviation (SD), spectrum skew, and spectrum kurtosis. In addition, the maximum amplitude frequency, and maximum amplitude of the maximum amplitude frequency were recorded at the middle 20 ms of the fricative. We include two Praat scripts with this submission. One extracts the measurements with no filtering in place. The other extracts the measurements after applying a high pass filter at 300 Hz. Both scripts extract spectral measurements based on the Nyquist frequency, 22,500 Hz, of the recordings.

The comparison of the measurements under these two analysis conditions (filter or no filter) is reported in Fig. 1, Fig. 2, Fig. 3, Fig. 4, Fig. 5, Fig. 6, Fig. 7 above. The two sets of measurements—with and without a high pass filter—are included in the data set submitted with this paper.

The R script included with the submission provides code for loading the data, removing outliers, and reproducing the plots in Fig. 1-7. We discarded extreme outliers, defined as tokens that were greater than three standard deviations from the mean of the high-pass-filtered spectrum CoG or the high-pass-filtered spectrum SD. This resulted in the loss of 35 tokens or 0.03% of the data. This is a slightly smaller number of outliers than are present if we calculated outliers based on the unfiltered data (there are 46 outliers, 0.05% of the data, in the unfiltered measurements).

## Ethics

The research activities that generated the data, involving non-evasive recording of speech, are excepted from Xihua University IRB. All participants in the study gave informed consent to be recorded and to have the recordings analysed for academic (non-commercial) purposes.

## CRediT Author Statement

**Dongmei Rao:** Conceptualization, Methodology, Investigation, Resources, Writing, Funding acquisition; **Jason Shaw:** Conceptualization, Methodology, Software, Formal analysis, Data Curation, Writing, Visualization; **Rikker Dockum:** Software, Resources.

## Declaration of Competing Interest

The authors declare that they have no known competing financial interests or personal relationships which have, or could be perceived to have, influenced the work reported in this article.

## Data Availability

Acoustic recordings of Zhongjiang Chinese non-coronal fricatives (Original data) (OSF).

## Appendix Supplementary materials

Image, application 1

Image, application 2

Image, application 3

Image, application 4
